# Supplementary material for: Sm-like protein Rof inhibits transcription termination factor ρ by binding site obstruction and conformational insulation
Source: Nat Commun. 2024 Apr 15;15:3186. doi: 10.1038/s41467-024-47439-6 (PMC11018626; doi:10.1038/s41467-024-47439-6)

Supplementary Information

Sm-like protein Rof inhibits transcription termination factor ρ by binding site obstruction and conformational insulation

Nelly Said[1], Mark Finazzo[2], Tarek Hilal[1,3], Bing Wang[2], Tim Luca Selinger[1], Daniela Gjorgjevikj[1], Irina Artsimovitch[2,*], Markus C. Wahl[1,4,*]

[1] Freie Universität Berlin, Institute of Chemistry and Biochemistry, Laboratory of Structural Biochemistry, Takustr. 6, D-14195 Berlin, Germany

[2] The Ohio State University, Department of Microbiology and Center for RNA Biology, Columbus, OH, USA

[3] Freie Universität Berlin, Institute of Chemistry and Biochemistry, Research Center of Electron Microscopy and Core Facility BioSupraMol, Fabeckstr. 36a, 14195 Berlin, Germany

[4] Helmholtz-Zentrum Berlin für Materialien und Energie, Macromolecular Crystallography, Albert-Einstein-Str. 15, D-12489 Berlin, Germany

* Correspondence to: artsimovitch.1@osu.edu; markus.wahl@fu-berlin.de

Supplementary Tables

Supplementary Table 1: IA227 mutations vs. RefSeq MG1655 GCF_000005845.2.

Nucleotide polymorphisms					
**Location**	**Gene**	**Reference**	**Mutation**	**Amino acid substitution**	**Gene function**
557602	folD	G	A	S47L	bifunctional methylenetetrahydrofolate dehydrogenase/methenyltetrahydrofolate cyclohydrolase
747956	ybgO	C	T	D343N	putative fimbrial protein
1907308	cspC	GTTTTGA	GTTTGA	cspC T43L + frameshift	transcription antiterminator and regulator of mRNA stability
2867461	rpoS	CTAA	CTATAA	V31*	RNA polymerase σ^S initiation factor
2984469	yqeF	G	A	A376V	putative acyltransferase
3031918	uacT	T	C	Y184Y	urate:H(+) symporter
3562445	glpD	TGGGTA	TGTA	W145C + frameshift	aerobic glycerol 3-phosphate dehydrogenase
3822476	spoT	C	A	A26E	bifunctional (p)ppGpp synthase/hydrolase
4121251	hslU	C	T	G166D	ATPase component of the HslVU protease
4296380	noncoding	ACCGCA	ACGCCGCA	none	
**Novel IS (insertion sequences) element insertions**					
**Location**	**Gene**	**IS type**	**Insertion disrupts protein**		**Gene function**
4123529	cytR	IS1	at residue ~310 (of 341)		DNA-binding transcriptional repressor
1873030	dgcJ	IS1	at residue ~330 (of 496)		diguanylate cyclase
4117981	glpF	IS30	at residue ~37 (of 281)		glycerol facilitator
2912721	relA	IS2	at residue ~311 (of 744)		GDP/GTP pyrophosphokinase
**Novel IS element deletions**					
**Location**	**Gene**	**IS type**			**Gene function**
257900-258675	crl	IS1I	IS1I present in the RefSeq genome is missing in IA227		RNAP σ^S holoenzyme assembly factor; activates many genes in the RpoS regulon

Supplementary Table 2: CryoEM data collection, refinement and validation statistics.

**Data collection and processing**					
	ρ_6-ADP-Rof$_5$	ρ_5-ADP-Rof$_4$	ρ_6-Rof$_5$	ρ_5-Rof$_4$	ρ-*rut* RNA
Microscope	FEI Titan Krios G3i				
Voltage [keV]	300				
Camera	Falcon 3EC				
Magnification (nominal)	96,000x				
Pixel size at detector [Å/pixel]	0.832	0.832	0.832	0.832	0.657
Total electron exposure [e^-/Å^2]	42	42	42	42	42
Exposure rate [e^-/pixel/s]	0.7	0.7	0.7	0.7	0.6
Frames collected during exposure	33	33	33	33	33
Defocus range [µm]	0.8 - 2	0.8 - 2	0.8 - 2	0.8 - 2	0.8 - 2
Automation software	EPU version 2.10				
Micrographs<br>Collected<br>Used	 4216<br>3982	 4216<br>3982	 1555<br>1521	 1555<br>1521	 3312<br>3273
Particle images<br>Total extracted<br>Final	 1,782,459<br>110,264	 1,782,459<br>293,952	 619,090<br>108,750	 619,090<br>285,647	 1,145,072<br>214,758
Point-group or helical symmetry parameters	C1	C1	C1	C1	C1
Resolution [Å]<br>Global<br>$FSC_{0.143}$[(a)] (unmasked/masked)<br>Local resolution range [Å^2]	 2.9<br>3.6/2.9<br>2.3 - 35	 2.7<br>3.3/2.7<br>1.8 - 35	 3.3<br>3.9/3.3<br>2.3 - 35	 3.04<br>3.5/3.04<br>2.4 - 35	 2.9<br>3.3 / 2.9<br>2.1 - 28
Map sharpening *B* factor/range [Å^2]	98.6	89.9	105.6	98.2	93.8
Map sharpening methods	local B-factor				
Refinement software<br>Package<br>Routine	 PHENIX version 1.20_4459<br>real.space.refine				
**Model composition**					
	ρ_6-ADP-Rof$_5$	ρ_5-ADP-Rof$_4$	ρ_6-Rof$_5$	ρ_5-Rof$_4$	ρ-*rut* RNA
Model composition<br>Non-H atoms<br>Protein residues<br>RNA residues<br>Mg^{2+} ions<br>ADP<br>BeF	 23.065<br>2.918<br>-<br>3<br>3<br>-	 19.124<br>2.418<br>-<br>-<br>3<br>-	 22.976<br>2.918<br>-<br>-<br>-<br>-	 19.039<br>2.418<br>-<br>-<br>-<br>-	 20.293<br>2.514<br>15<br>6<br>6<br>6
**Model refinement**					
Model-Map scores<br>CC[(b)] (mask)<br>CC (volume)	 0.88<br>0.88	 0.90<br>0.90	 0.83<br>0.83	 0.82<br>0.82	 0.88<br>0.88
FSC model = 0.5 [Å]<br>(masked/unmasked)	3.05/3.04	2.85/2.88	3.38/3.47	3.20/3.25	2.94/2.97
Average grouped B factors [Å^2]<br>Overall<br>Protein<br>RNA<br>Mg^{2+} ions<br>ADP<br>BeF	 145<br>145<br>-<br>129<br>167<br>-	 113<br>113<br>-<br>-<br>137<br>-	 173<br>173<br>-<br>-<br>-<br>-	 87<br>87<br>-<br>-<br>-<br>-	 101<br>102<br>96<br>63<br>73<br>86
Rmsd[(c)] from ideal values<br>Bond lengths [Å]<br>Bond angles [°]	 0.003<br>0.421	 0.003<br>0.429	 0.003<br>0.708	 0.003<br>0.640	 0.004<br>0.754
**Validation[(d)]**					
MolProbity score	1.49	1.66	1.63	1.60	1.93
CaBLAM outliers [%]	0.70	0.84	0.70	0.76	0.48
Clashscore	5.61	6.93	13.07	12.1	11.23

Poor rotamers [%]	1.75	2.35	0.48	0.14	3.02
Cβ deviations	0.0	0.0	0.0	0.0	0.0
EMRinger score	2.99	3.39	1.32	1.18	2.96
Ramachandran plot					
Favored [%]	98.83	98.62	98.20	98.33	98.56
Allowed [%]	1.17	1.38	1.76	1.67	1.44
Outliers [%]	0.0	0.0	0.03	0.0	0.0
Ramachandran *Z*-score (rmsd)					
Overall	1.52 (0.16)	1.31 (0.17)	0.48 (0.16)	0.96 (0.17)	0.98 (0.17)
Helices	1.65 (0.15)	1.30 (0.17)	1.09 (0.16)	1.41 (0.17)	1.41 (0.16)
Sheets	0.56 (0.24)	0.59 (0.27)	0.13 (0.26)	0.12 (0.26)	0.04 (0.26)
Loops	0.74 (0.18)	0.77 (0.20)	-0.26 (0.17)	0.23 (0.20)	0.23 (0.20)
Data deposition					
Reconstruction (EMDB)	EMD-17874	EMD-17876	EMD-17875	EMD-17877	EMD-17870
Coordinates (PDB)	8PTM	8PTO	8PTN	8PTP	8PTG

[a] FSC, Fourier shell correlation
[b] CC, correlation coefficient
[c] Rmsd, root-mean-square deviation
[d] Using MolProbity[1]

Supplementary Table 3: Oligonucleotides and plasmids used in this work.

**Oligonucleotides for cryoEM and interaction studies**		
rut RNA	GGGAUAACCCCGCUCUUACACAUUCCAGCCCUGAAAAAGGGCAUCAAAUUAAACCACACCUAUGGUGUAUGUCAAAUUAAACCACA	[2]
rut-less RNA	ACACCUGGCGUGUGGC	This work
DNA_antisense_scaffold	GTTATCCGCTCACAATGCCACACGCGCTGCTCGGCCGTTATTCGCAGCCC	[2]
DNA_sense_scaffold	GGGCTGCGAATAACGGCCGAGCAGCGTAGCATTACTTGTGAGCGGATAAC	[2]
**Oligonucleotides for template preparation**		
λP_R_UP	CGTTAAATCTATCACCGCAAGG	IA lab stock #17
λP_R_DN	CAGTTCCCTACTCTCGCATG	IA lab stock #256
**Plasmids for protein production and in vitro transcription**		
pIA267	λ P_R promoter – C-less A26 ITR - λ *tR1* terminator template	[3]
pIA1352	λ P_R promoter – C-less A26 ITR – Salmonella *siiE* template	This work
pIA586	*E. coli* σ^{70} expression	[2]
pVS10	*E. coli* core RNAP expression; His_6-tagged β' subunit	[2]
pET24b-*rho*	*E. coli rho* expression	[4]
pETM11-*rho*	*E. coli rho* expression	[5]
pETM11-*rho* [R88E]	*E. coli rho* expression [R88E]	This work
pETM11-*rho* [F89S]	*E. coli rho* expression [F89S]	This work
pETM11-*rho* [K115E]	*E. coli rho* expression [K115R]	This work
pNS-100	*E. coli rof* expression; N-terminal His_6-TEV-tag	This work
pNS-100b	*E. coli rof* expression [Y13A]; N-terminal His_6-TEV-tag	This work
pNS-100c	*E. coli rof* expression [D14A]; N-terminal His_6-TEV-tag	This work
pNS-100d	*E. coli rof* expression [E17A]; N-terminal His_6-TEV-tag	This work
**Plasmids for in vivo assays; P_{trc} – *INSERT***		
pTrc99c	none	[6]
pIA1488	– *E. coli* rof^{wt}	This work
pIA1530	– *E. coli rof* [Y13A]	This work
pIA1532	– *E. coli* His_6-*rof*	This work
pIA1544	– *E. coli* rof^{wt} [HA@57]	This work
pIA1545	– *E. coli rof* [C10S]	This work
pIA1557	– *E. coli rof* [Y13A;HA@57]	This work
pIA1574	– *E. coli rof* [K47A]	This work
pIA1575	– *E. coli rof* [R46A]	This work
pIA1576	– *E. coli rof* [N48A]	This work
pIA1577	– *E. coli rof* [D14A]	This work
pIA1589	– *E. coli rof* [E17K]	This work
pIA1615	– *E. coli rof* [D14A;HA@57]	This work

pIA1616	– *E. coli rof* [E17K;HA@57]	This work
pIA1617	– *E. coli rof* [R46A;HA@57]	This work
pIA1618	– *E. coli rof* [K47A;HA@57]	This work

Supplementary Figures

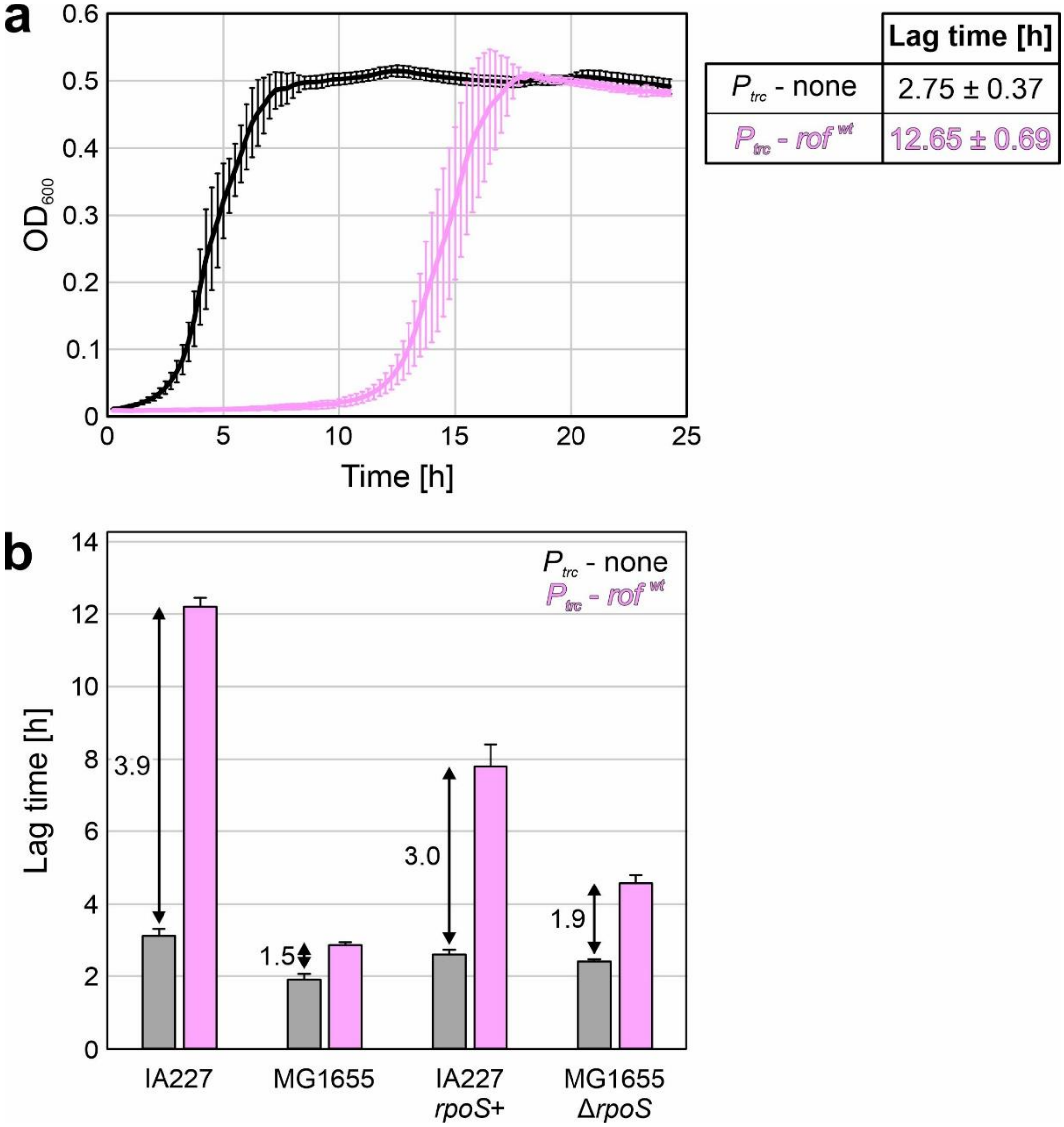

Supplementary Fig. 1: Rof effect on cell growth.

a, Rof expression extends the lag time but does not alter the exponential growth phase. Cells transformed with a plasmid expressing wt Rof (violet) or an empty vector (black) were grown in MOPS EZ rich defined media supplemented with carbenicillin overnight at 32 °C, diluted into fresh media supplemented with carbenicillin and 1 mM IPTG, and grown in a microplate reader at 32 °C for 24 hours. Cell growth was monitored by OD_{600} measured every 15 min. The data from four

biological replicates were fit to the modified Gompertz equation[7]. Source data are provided as a Source Data file.

b, Rof-induced toxicity is exacerbated in the absence of *rpoS*. Plasmids from panel (**a**) were transformed into selected strains, IA227 [*rpoS*º], MG1655 [*rpoS*⁺], IA227 with *rpoS*⁺ restored by P1 transduction of a linked Δ*ygbN*::Kn from JW2710 (Keio collection), and MG1655 Δ*rpoS* constructed by P1 transduction of *rpoS*::Kn from JW5437. Strains were grown as in panel (**a**); the data from five biological replicates were used to calculate the lag times. Double arrows/numbers, fold increase in lag time upon *rof* expression. Source data are provided as a Source Data file.

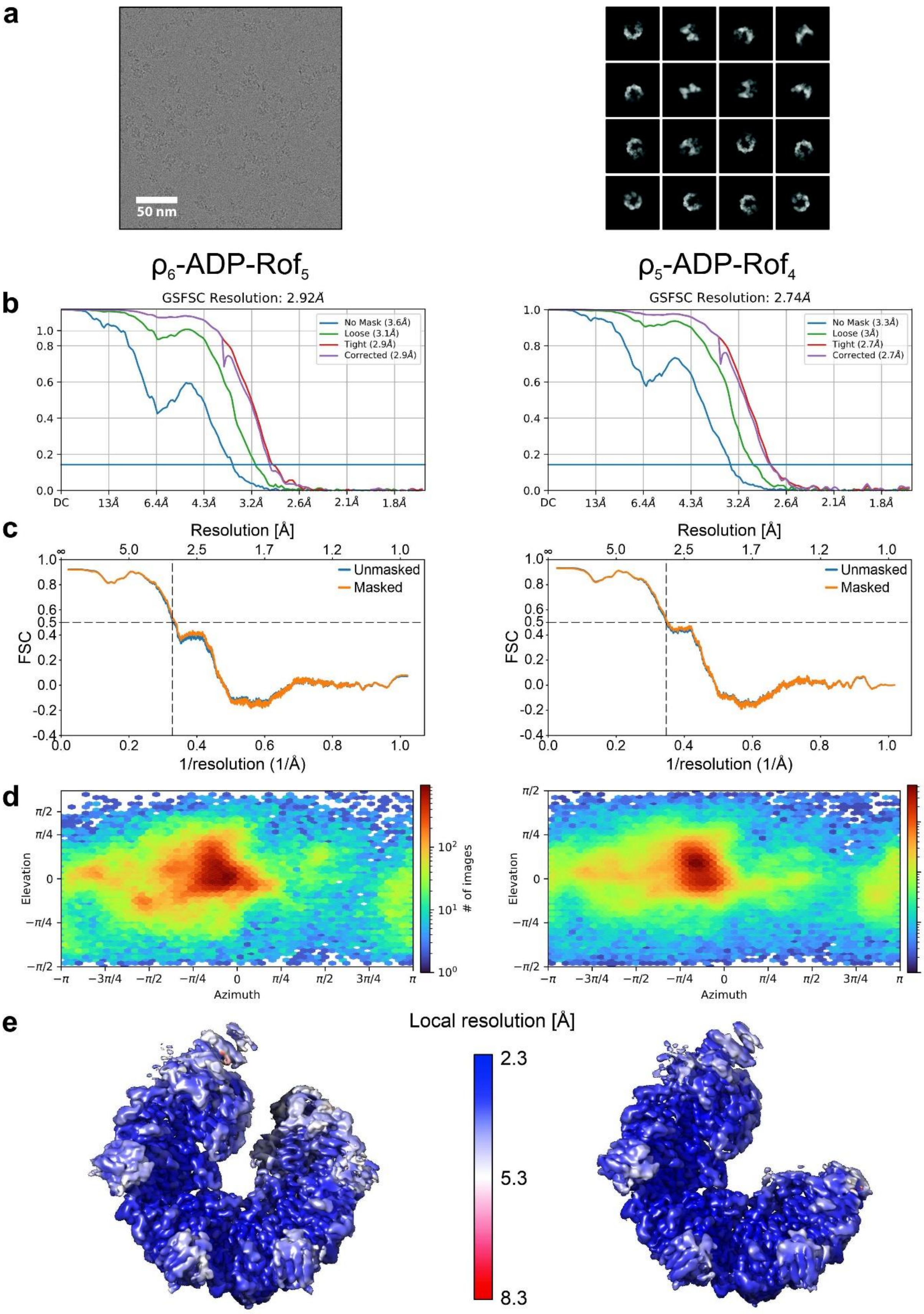

a
50 nm
ρ6-ADP-Rof5
ρ5-ADP-Rof4
b
GSFSC Resolution: 2.92Å
No Mask (3.6Å)
Loose (3.1Å)
Tight (2.9Å)
Corrected (2.9Å)
GSFSC Resolution: 2.74Å
No Mask (3.3Å)
Loose (3Å)
Tight (2.7Å)
Corrected (2.7Å)
c
Resolution [Å]
FSC
Unmasked
Masked
1/resolution (1/Å)
d
Elevation
Azimuth
# of images
e
Local resolution [Å]
2.3
5.3
8.3

Supplementary Fig. 2: CryoEM/SPA analysis of ρ-ADP-Rof complexes.

a, Representative cryoEM micrograph (left; scale bar, 50 nm) and selected class averages (right) of ρ-ADP-Rof complexes after reference-free 2D classification.

b, Gold-standard Fourier shell correlation plots after NU refinement of the ρ_6-ADP-Rof_5 (left) and ρ_5-ADP-Rof_4 (right) reconstructions.

c, Model-to-map cross resolution (FSC 0.5) plots as determined during phenix real space refinement of the ρ_6-ADP-Rof_5 (left) and ρ_5-ADP-Rof_4 (right) structures before (unmasked) and after (masked) applying a soft mask. The resolutions at the FSC 0.5 threshold are indicated by dashed lines.

d, Viewing direction distributions of the ρ_6-ADP-Rof_5 (left) and ρ_5-ADP-Rof_4 (right) reconstructions.

e, Top views of the cryoEM reconstructions of the ρ_6-ADP-Rof_5 (left) and ρ_5-ADP-Rof_4 (right) complexes, colored according to the local resolution.

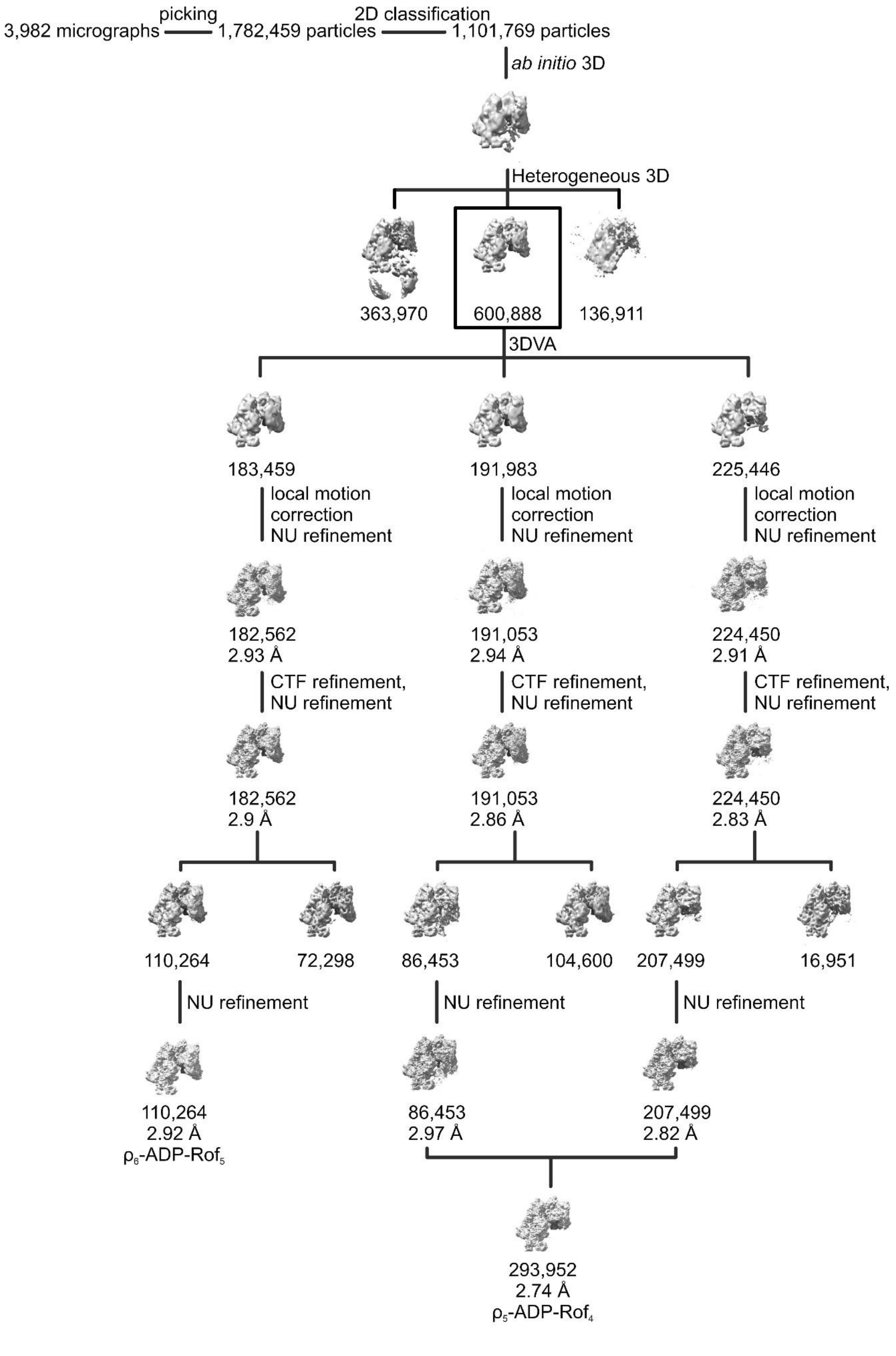
picking
2D classification
3,982 micrographs
1,782,459 particles
1,101,769 particles
ab initio 3D
Heterogeneous 3D
363,970
600,888
136,911
3DVA
183,459
191,983
225,446
local motion correction NU refinement
local motion correction NU refinement
local motion correction NU refinement
182,562 2.93 Å
191,053 2.94 Å
224,450 2.91 Å
CTF refinement, NU refinement
CTF refinement, NU refinement
CTF refinement, NU refinement
182,562 2.9 Å
191,053 2.86 Å
224,450 2.83 Å
110,264
72,298
86,453
104,600
207,499
16,951
NU refinement
NU refinement
NU refinement
110,264 2.92 Å ρ_6-ADP-Rof_5
86,453 2.97 Å
207,499 2.82 Å
293,952 2.74 Å ρ_5-ADP-Rof_4

Supplementary Fig. 3: ρ-ADP-Rof cryoEM data refinement.

From 3,982 micrographs a total of 1,782,459 particle images were initially picked and subjected to reference-free 2D classification. 1,101,769 particle images were selected and used for ab initio 3D reconstruction to generate an initial reference for heterogeneous 3D refinement. A subset of 600,888 particle images was selected for further classification by 3D variability analysis that already revealed existence of ρ_6-ADP-Rof_5 and ρ_5-ADP-Rof_4 states. Local motion correction followed by CTF refinement, heterogeneous 3D refinement and subsequent NU refinement was applied to isolate the final particle sets and generate reconstructions of the ρ_6-ADP-Rof_5 and ρ_5-ADP-Rof_4 particles at global resolutions of 2.92 Å and 2.74 Å, respectively.

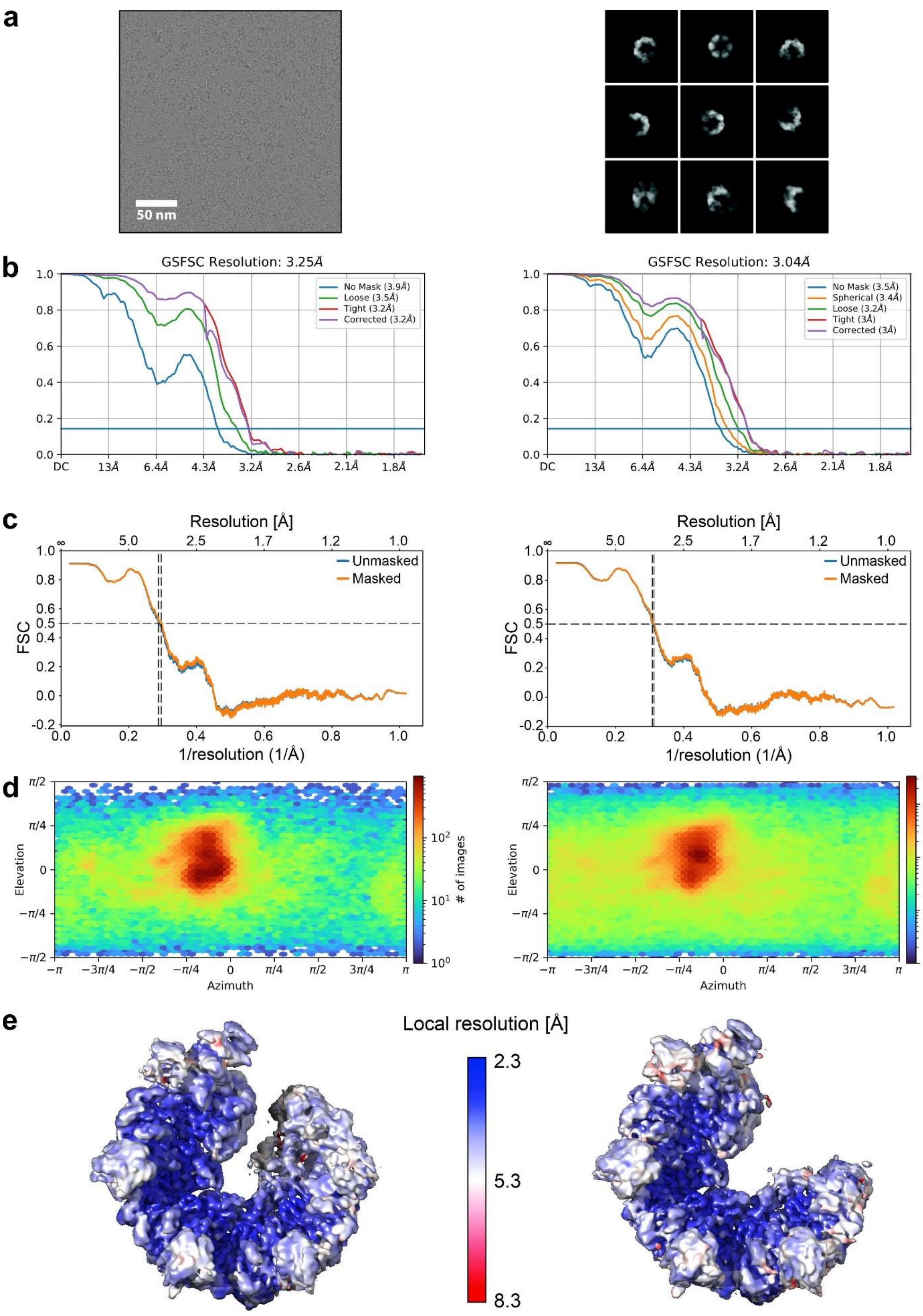
a
50 nm
b
GSFSC Resolution: 3.25Å
No Mask (3.9Å)
Loose (3.5Å)
Tight (3.2Å)
Corrected (3.2Å)
GSFSC Resolution: 3.04Å
No Mask (3.5Å)
Spherical (3.4Å)
Loose (3.2Å)
Tight (3Å)
Corrected (3Å)
DC 13Å 6.4Å 4.3Å 3.2Å 2.6Å 2.1Å 1.8Å
c
Resolution [Å]
FSC
Unmasked
Masked
1/resolution (1/Å)
d
Elevation
Azimuth
# of images
e
Local resolution [Å]
2.3
5.3
8.3

Supplementary Fig. 4: CryoEM/SPA analysis of ρ-Rof complexes.

a, Representative cryoEM micrograph (left; scale bar, 50 nm) and selected class averages (right) of ρ-Rof complexes after reference-free 2D classification.

b, Gold-standard Fourier shell correlation plots after NU refinement of the $ρ_6$-Rof_5 (left) and $ρ_5$-Rof_4 (right) reconstructions.

c, Model-to-map cross resolution (FSC 0.5) plots as determined during phenix real space refinement of the $ρ_6$-Rof_5 (left) and $ρ_5$-Rof_4 (right) structures before (unmasked) and after (masked) applying a soft mask. The resolutions at the FSC 0.5 threshold are indicated by dashed lines.

d, Viewing direction distributions of the $ρ_6$-Rof_5 (left) and $ρ_5$-Rof_4 (right) reconstructions.

e, Top views of the cryoEM reconstructions of the $ρ_6$-Rof_5 (left) and $ρ_5$-Rof_4 (right) complexes, colored according to the local resolution.

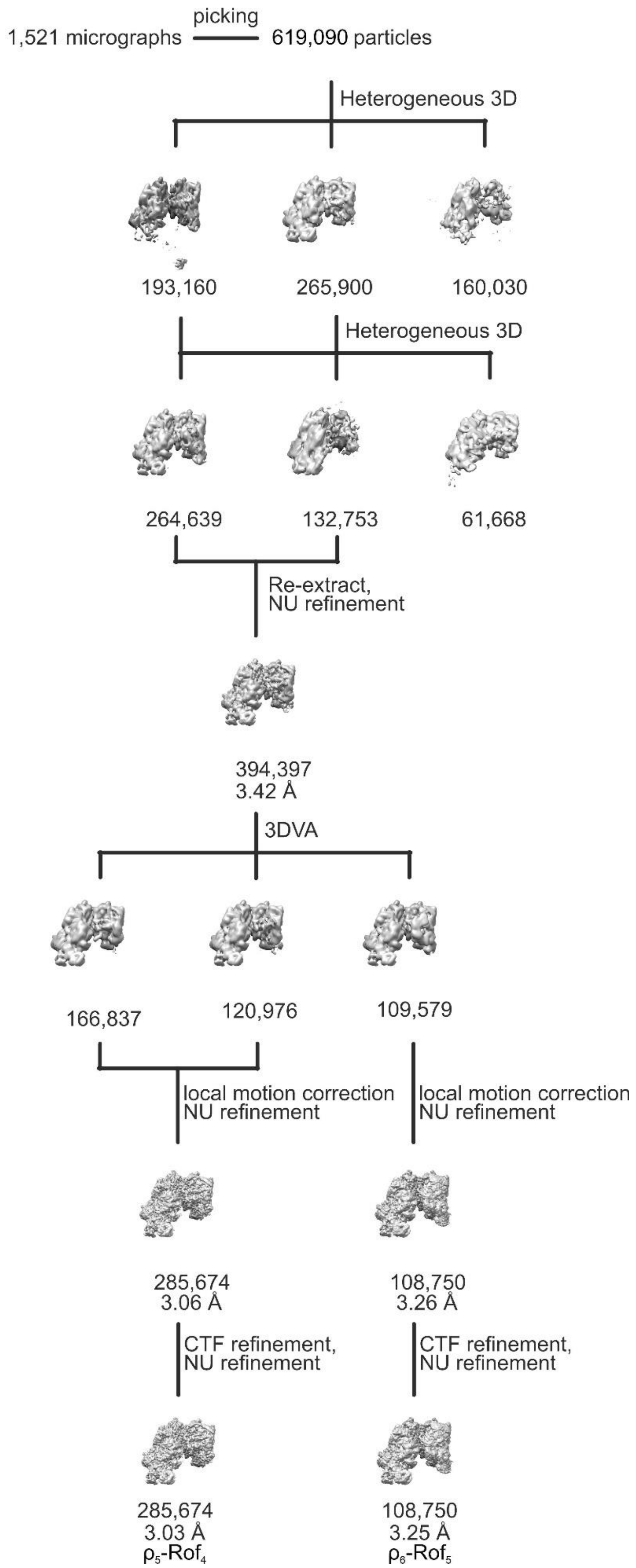

1,521 micrographs
picking
619,090 particles
Heterogeneous 3D
193,160
265,900
160,030
Heterogeneous 3D
264,639
132,753
61,668
Re-extract,
NU refinement
394,397
3.42 Å
3DVA
166,837
120,976
109,579
local motion correction
NU refinement
local motion correction
NU refinement
285,674
3.06 Å
108,750
3.26 Å
CTF refinement,
NU refinement
CTF refinement,
NU refinement
285,674
3.03 Å
ρ_5-Rof_4
108,750
3.25 Å
ρ_6-Rof_5

Supplementary Fig. 5: ρ-Rof cryoEM data refinement.

From 1,521 micrographs a total of 619,090 particles were initially picked and subjected to iterative heterogeneous 3D refinement cycles using the ρ-ADP-Rof reconstructions as reference. 394,397 particle images were selected for re-extraction and further classification by 3D variability analysis. Local motion correction of the selected 285,674 particle images, followed by CTF refinement and NU refinement yielded a reconstruction at a global resolution of 3.03 Å.

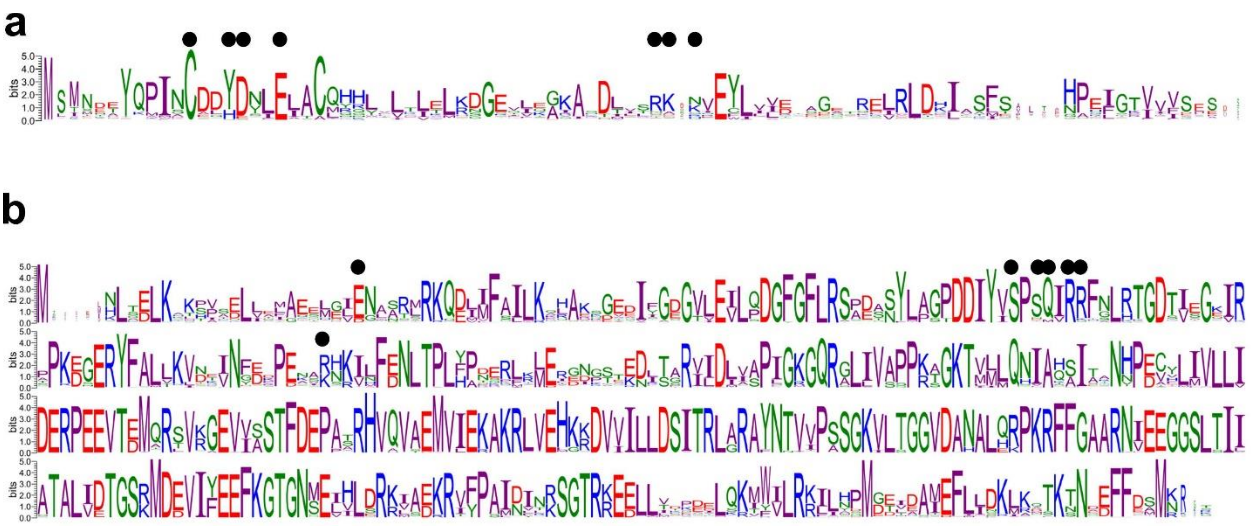

Supplementary Fig. 6: Rof and ρ conservation.

a, Rof conservation.

b, ρ conservation. In (**a**) and (**b**), the ρ-Rof interface residues revealed by the complex structures are indicated by black dots. Sequence logos were generated by WebLogo (version 3.7.8).

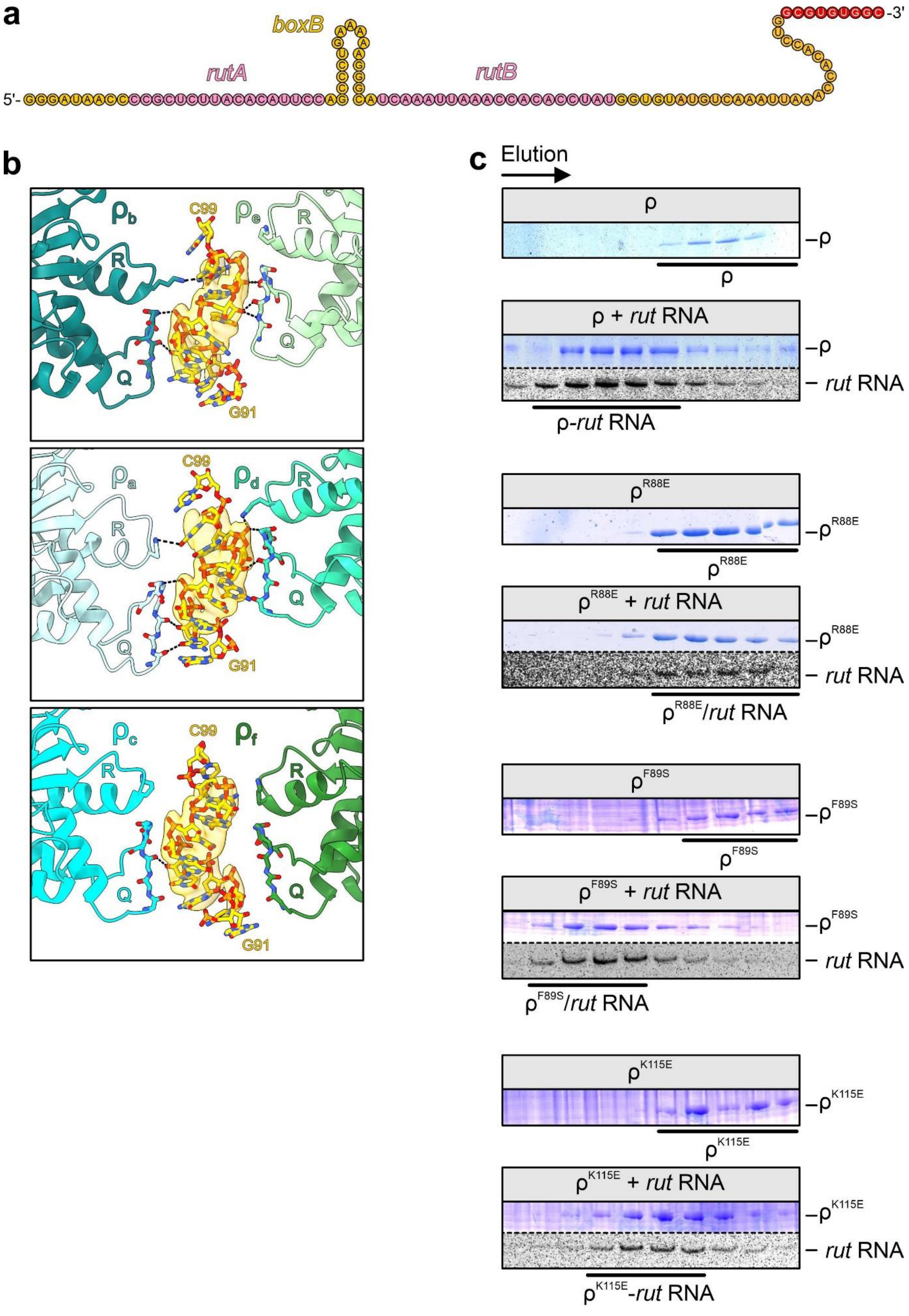
a
boxB
rutA
rutB
5'-
-3'
b
ρb
ρe
C99
G91
R
Q
ρa
ρd
ρc
ρf
c
Elution
ρ
–ρ
ρ + rut RNA
– rut RNA
ρ-rut RNA
ρR88E
–ρR88E
ρR88E + rut RNA
ρR88E/rut RNA
ρF89S
–ρF89S
ρF89S + rut RNA
ρF89S/rut RNA
ρK115E
–ρK115E
ρK115E + rut RNA
ρK115E-rut RNA

Supplementary Fig. 7: ρ-RNA interactions.

a, *Rut* RNA used in this study; *rutA*, *rutB* (light red) and the *boxB* hairpin are labeled. Nine 3'-nucleotides modeled at the ρ SBS, red.

b, ρ binds SBS RNA via the Q- and R-loops. RNA, Q-loop residues (281-287) and R-loop K326 of the ρ subunits are depicted as sticks. The RNA oriented with the 5'-end at the bottom and the 3'-end at the top. The region of the cryoEM reconstruction covering the six nucleotides at the center of the ρ ring is shown as a semi-transparent surface. Two/one residue/s 5'/3' of this region are additionally defined by weaker density, not visible at the present contour level. The three panels show the three pairs of ρ subunits positioned opposite to each other around the RNA. Most of the contacts are not sequence-specific and are mediated by backbone interactions of both, RNA and ρ.

c, Analytical SEC runs, monitoring the binding of ρ variants to *rut* RNA. For each run, the same fractions were analyzed by SDS-PAGE (protein) and 8 M urea-PAGE (RNA). Gray boxes above the gels indicate the ρ variant or the ρ variant/*rut* RNA mixture analyzed. Experiments were performed three times independently with similar results. Source data are provided at the end of the Supplementary Information file.

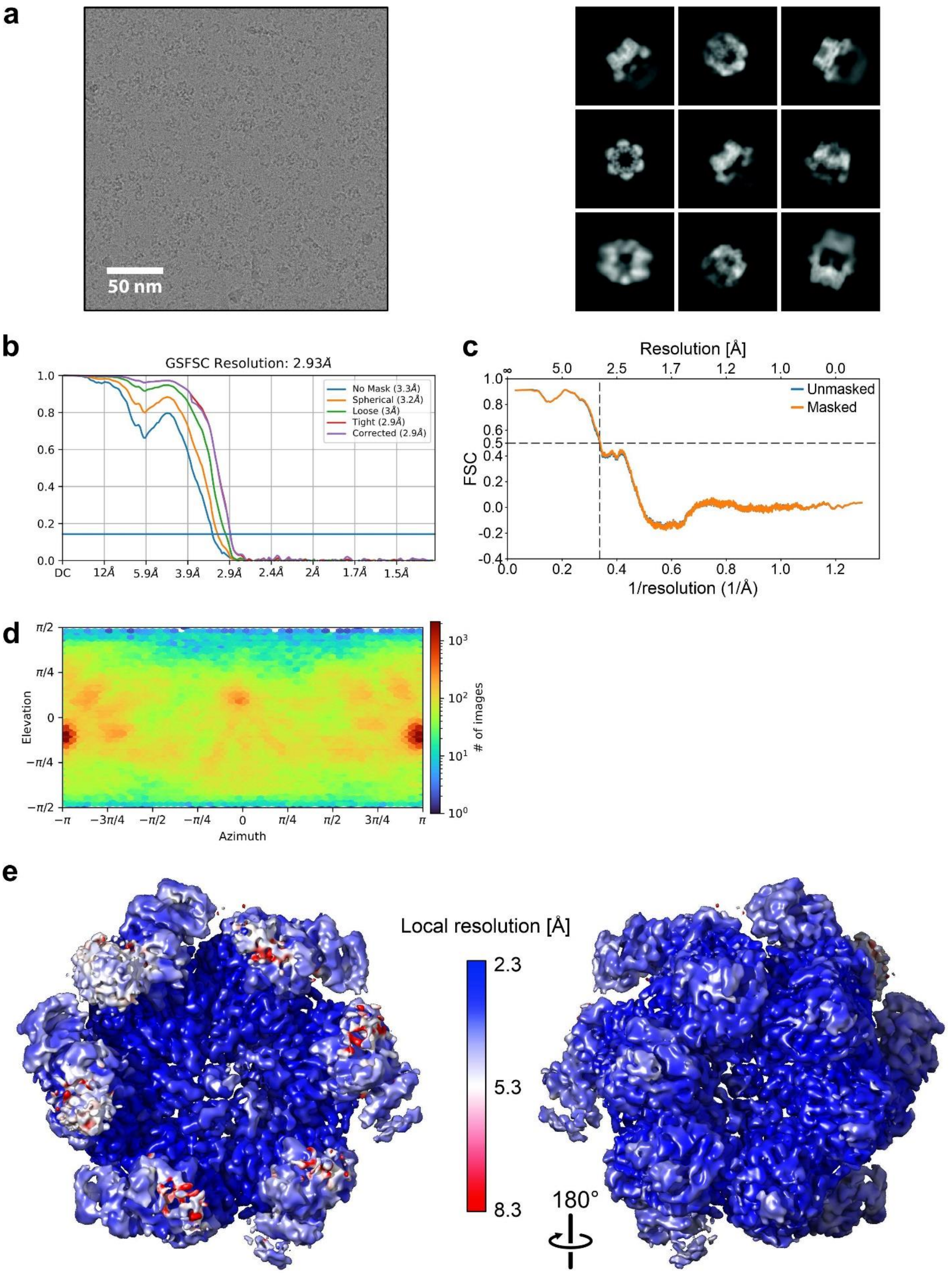
a
50 nm
b
GSFSC Resolution: 2.93Å
No Mask (3.3Å)
Spherical (3.2Å)
Loose (3Å)
Tight (2.9Å)
Corrected (2.9Å)
DC
12Å
5.9Å
3.9Å
2.9Å
2.4Å
2Å
1.7Å
1.5Å
c
Resolution [Å]
Unmasked
Masked
FSC
1/resolution (1/Å)
d
Elevation
Azimuth
# of images
e
Local resolution [Å]
2.3
5.3
8.3
180°

Supplementary Fig. 8: CryoEM/SPA analysis of ρ-*rut* RNA complexes.

a, Representative cryoEM micrograph (left; scale bar, 50 nm) and selected class averages (right) of ρ-*rut* RNA complexes after reference-free 2D classification.

b, Gold-standard Fourier shell correlation plot after NU refinement of the ρ-*rut* RNA reconstruction.

c, Model-to-map cross resolution (FSC 0.5) plots as determined during phenix real space refinement of the ρ-*rut* RNA structure before (unmasked) and after (masked) applying a soft mask. The resolutions at the FSC 0.5 threshold are indicated by dashed lines.

d, Viewing direction distribution of the ρ-*rut* RNA reconstruction.

e, Top (left) and bottom (right) views of the cryoEM reconstruction of the ρ-*rut* RNA complex, colored according to the local resolution.

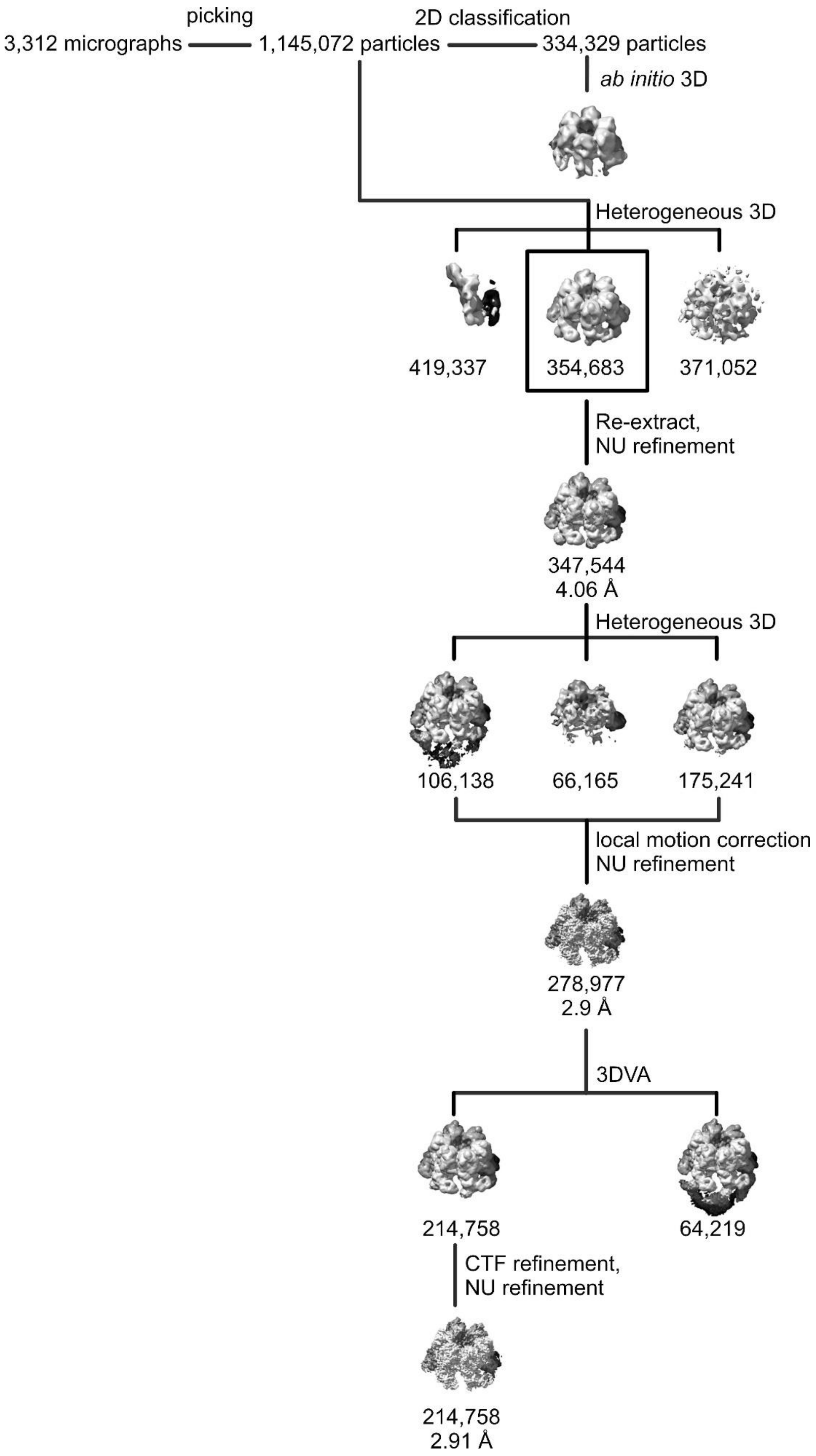
picking
3,312 micrographs
1,145,072 particles
2D classification
334,329 particles
ab initio 3D
Heterogeneous 3D
419,337
354,683
371,052
Re-extract,
NU refinement
347,544
4.06 Å
Heterogeneous 3D
106,138
66,165
175,241
local motion correction
NU refinement
278,977
2.9 Å
3DVA
214,758
64,219
CTF refinement,
NU refinement
214,758
2.91 Å

Supplementary Fig. 9: ρ-*rut* RNA cryoEM data refinement.

From 3,312 micrographs a total of 1,145,072 particles were initially picked and subjected to reference-free 2D classification. 334,329 particle images were selected and used for ab initio 3D reconstruction to generate an initial reference for heterogeneous 3D refinement of the entire dataset. A subset of 354,683 particle images was selected for further classification by heterogeneous 3D refinement followed by local motion correction and additional classification by 3D variability analysis. CTF refinement and subsequent NU refinement of the finally selected 214,758 particle images yielded a 3D reconstruction at 2.91 Å resolution.

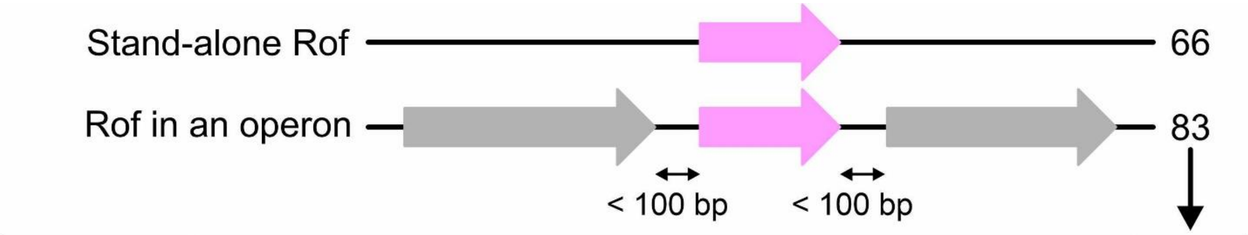

COG categories	Annotation	#
E	Amino acid transport and metabolism	1
G	Carbohydrate transport and metabolism	6
H	Coenzyme transport and metabolism	4
J	Translation, ribosomal structure and biogenesis	14
M	Cell wall/membrane/envelope biogenesis	3
O	Posttranslational modification, protein turnover, chaperones	8
P	Inorganic ion transport and metabolism	5
Q	Secondary metabolites biosynthesis, transport and catabolism	11
R	General function prediction only	6
S	Function unknown	12
T	Signal transduction mechanisms	4

Supplementary Fig. 10: Gene neighbors of *rof*.

Analysis of *rof* gene neighbors collected from TREND[8] (Supplementary Dataset 1d) shows that slightly fewer than half (66 out of 149) of the *rof* genes are not located within operons; the distance to the nearest co-directional neighbor is more than 100 bp. For those *rof* genes that are located within a hypothetical operon (83 out of 149), we assigned clusters of orthologous groups (COG) [9] to the closest neighbor of *Vibrionaceae rof*, located within 100 bp of the *rof* gene and translated from the same strand. Among 11 identified COG categories, two (J and Q), associated with the ribosome biogenesis and with the production of secondary metabolites functions, are somewhat overrepresented among the *rof* nearest neighbors.

Supplementary References

1. Prisant, M.G., Williams, C.J., Chen, V.B., Richardson, J.S. & Richardson, D.C. New tools in MolProbity validation: CaBLAM for CryoEM backbone, UnDowser to rethink "waters," and NGL Viewer to recapture online 3D graphics. *Protein Sci* **29**, 315-329 (2020).
2. Svetlov, V., Belogurov, G.A., Shabrova, E., Vassylyev, D.G. & Artsimovitch, I. Allosteric control of the RNA polymerase by the elongation factor RfaH. *Nucleic Acids Res* **35**, 5694-5705 (2007).
3. Artsimovitch, I. & Landick, R. The transcriptional regulator RfaH stimulates RNA chain synthesis after recruitment to elongation complexes by the exposed nontemplate DNA strand. *Cell* **109**, 193-203 (2002).
4. Lawson, M.R. et al. Mechanism for the Regulated Control of Bacterial Transcription Termination by a Universal Adaptor Protein. *Mol Cell* **71**, 911-922 (2018).
5. Krupp, F. et al. Structural Basis for the Action of an All-Purpose Transcription Anti-termination Factor. *Mol Cell* **74**, 143-157 e145 (2019).
6. Amann, E., Ochs, B. & Abel, K.-J. Tightly regulated tac promoter vectors useful for the expression of unfused and fused proteins in Escherichia coli. *Gene* **69**, 301-315 (1988).
7. Zwietering, M.H., Jongenburger, I., Rombouts, F.M. & van 't Riet, K. Modeling of the bacterial growth curve. *Appl Environ Microbiol* **56**, 1875-1881 (1990).
8. Gumerov, V.M. & Zhulin, I.B. TREND: a platform for exploring protein function in prokaryotes based on phylogenetic, domain architecture and gene neighborhood analyses. *Nucleic Acids Res* **48**, W72-W76 (2020).
9. Galperin, M.Y. et al. COG database update: focus on microbial diversity, model organisms, and widespread pathogens. *Nucleic Acids Res* **49**, D274-D281 (2021).

Source Data

Supplementary Fig. 7c

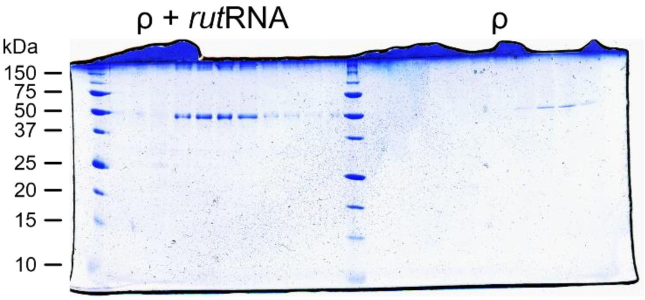

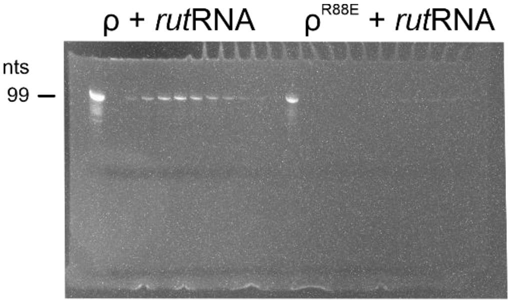

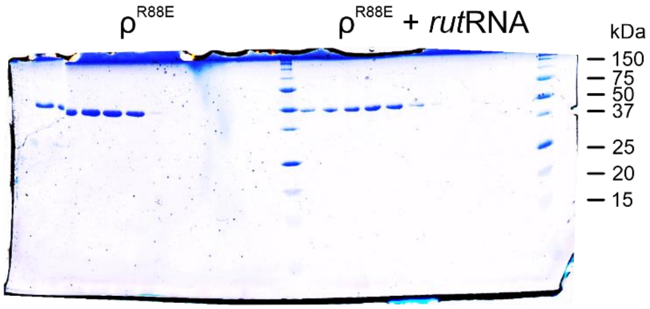

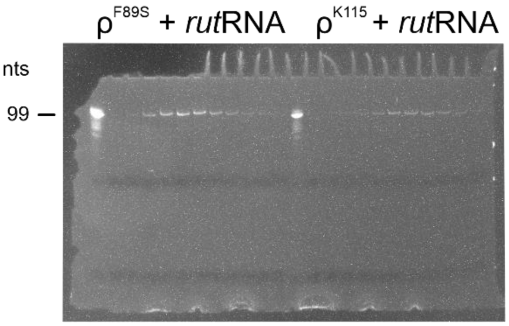

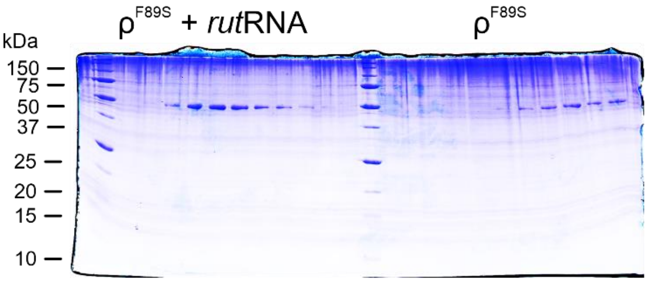

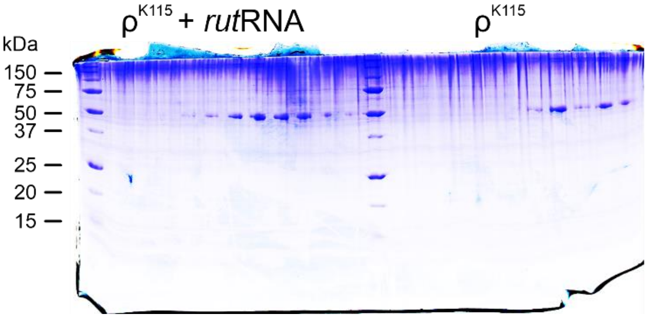

Supplement: Supplementary file 1 — Supplementary Information [file 41467_2024_47439_MOESM1_ESM.pdf]
