# Supplementary material for: Sm-like protein Rof inhibits transcription termination factor ρ by binding site obstruction and conformational insulation
Source: Nat Commun. 2024 Apr 15;15:3186. doi: 10.1038/s41467-024-47439-6 (PMC11018626; doi:10.1038/s41467-024-47439-6)
Supplement: Supplementary file 3 — Description of Additional Supplementary Files [file 41467_2024_47439_MOESM3_ESM.pdf]

Description of Additional Supplementary Files

File name: Supplementary Data 1
Description: Rof conservation analysis.
a, Rof distribution in families of Pseudomonadota, related to Fig. 1d. The fraction of genomes containing *rof* was estimated in Annotree.
b, *Rof* and *yaeP* overlapping (Fig. 8e) were investigated for genomes harboring both of them in *Enterobacteriaceae*.
c, *Rof* and *yaeP* overlapping (Fig. 8e) were investigated for genomes harboring both of them in *Vibrionaceae*.
d, *Vibrionaceae rof* genomic context was collected from TREND. Note that operon ID is not continuous, which is generated by TREND. An operon is defined if *rof* has a gene neighbor that is located on the same strand and within 100 bp.
e, Rof representatives for Supplementary Fig. 6a.
f, ρ representatives for Supplementary Fig. 6b.
g, *Enterobacteriaceae* representatives for Fig. 8c.
h, *Vibrionaceae* representatives for Fig. 8c.
